# Regional anesthesia and lipid resuscitation for local anesthetic systemic toxicity in China: results of a survey by the orthopedic anesthesia group of the chinese society of anesthesiology

**DOI:** 10.1186/s12871-015-0163-0

**Published:** 2016-01-04

**Authors:** Mao Xu, Shanliang Jin, Zhengqian Li, Xuzhong Xu, Xiuli Wang, Lan Zhang, Zeguo Feng, Buwei Yu, Jin Liu, Xiangyang Guo

**Affiliations:** 1 Department of Anesthesiology, Peking University Third Hospital, No. 49, North Garden Street, Haidian District, Beijing, 100191 China; 2 Department of Anesthesiology, No 3 People’s Hospital Affiliated to Shanghai Jiaotong University School of Medicine, Shanghai, China; 3 Department of Anesthesiology, the First Affiliated Hospital of Wenzhou Medical University, Zhejiang, China; 4 Department of Anesthesiology, the Third Hospital of Hebei Medical University, Hebei, China; 5 Department of Anesthesiology, Sichuan Orthopedic Hospital, Sichuan, China; 6 Anesthesia and Operation Center, Chinese PLA General Hospital and Medical School of Chinese PLA, Beijing, China; 7 Department of Anesthesiology, Ruijin Hospital, School of Medicine, Shanghai Jiaotong University, Shanghai, China; 8 Department of Critical Medicine and Anesthesiology, West China Hospital, Sichuan University, Sichuan Province, China

**Keywords:** Local anesthetic systemic toxicity, Lipid rescue therapy, Survey

## Abstract

**Background:**

Intravenous lipid emulsions have been introduced for the management of patients with Local Anesthetic Systemic Toxicity (LAST). These emulsions have been stated as a first-line treatment in the guidelines of several international anesthesia organizations. Nevertheless, the adoption of lipid rescue therapy by Chinese practitioners remains unknown. We, therefore, evaluated the current approaches to treat LAST and the use of lipid rescue therapy among anesthesiologists in China.

**Methods:**

In September 2013, a 23-question survey on regional anesthesia practice and availability of lipid emulsions was sent by e-mail to directors or designated individuals at 41 academic anesthesiology departments listed by the orthopedic anesthesia group of the Chinese Society of Anesthesiology.

**Results:**

Responses were received from 36 of the 41 (88 %) anesthesiology departments. To simplify the analysis, responses were divided into two groups according to the annual percentage of patients who received regional anesthesia (RA) for orthopedic anesthesia: 14 departments (39 %) with high-utilization (≥50 %) and 22 departments (61 %) low-utilization (<50 %) of RA. Ropivacaine and bupivacaine were the common drugs used for RA, which were independent of RA utilization. Interestingly, ultrasound-guided techniques were much more frequently used in low-utilization institutions than in high-utilization institutions (*P* = 0.025). Lipid emulsion was readily available in 8 of the 36 (22 %) responding institutions, with 7 of the other 28 (25 %) institutions planning to stock lipid emulsion. No differences in lipid availability and storage plans were observed between high- and low-utilization institutions. Lipid resuscitation was performed in five of the eight departments that had lipid emulsion. Eleven patients were successfully resuscitated and one was not.

**Conclusion:**

Lipid emulsion is not widely available in China to treat LAST resulted from RA for orthopedic patients. Efforts are required to promote lipid rescue therapy nationwide.

**Trial registration:**

Chinese Clinical Trail Registry (Registration number # ChiCTR-EOR-15006960; Date of Retrospective Registration on August 23rd, 2015) http://www.chictr.org.cn/showproj.aspx?proj=11703.

**Electronic supplementary material:**

The online version of this article (doi:10.1186/s12871-015-0163-0) contains supplementary material, which is available to authorized users.

## Background

The increased use of regional anesthesia (RA) have increased the incidence of local anesthetic systemic toxicity (LAST), which has been reported to range from 7.5 to 20 per 10,000 peripheral nerve blocks (PNBs) and at about 4 per 10,000 epidural blocks [1]. Although it is rare, LAST can be lethal. Many case reports [2–5] and animal studies [6, 7] have suggested that lipid emulsion infusion is effective in reversing LAST, resulting in the use of lipid rescue therapy (LRT) during resuscitation.

LRT has been explicitly supported by the Association of Anesthetists of Great Britain and Ireland (AAGBI), the American Society of Regional Anesthesia (ASRA), and the American Heart Association (AHA) guidelines on LAST treatment [8–10]. In addition, the American College of Medical Toxicology (ACMT) has issued interim aguidelines for the use of LRT in conditions other than LAST [11]. Similar national guidelines have not yet been introduced, but are under consideration, in China.

In 2006, a questionnaire study documented contemporary practice strategies among academic anesthesiology departments in the United States [12]. Two other national surveys, one in the United Kingdom in 2007 [13] and the other in the United States in 2011 [14], have assessed the availability of lipid emulsion in obstetric anesthesia units. Similarly, the availability of and use of lipid rescue therapy in England and Wales was also surveyed in 2009 [15]. To date, however, no comparable survey has been completed in China. The aim of this study was to evaluate the current status of RA practices and the adoption of LRT among anesthesiologists in China.

## Methods

This study was approved by the independent ethical committee of Peking University Third Hospital, Beijing, China (Approval No. 2013046). It was also registered with Chinese Clinical Trial Registry under the number ChiCTR-EOR-15006960. The approved questionnaire was developed by three of the investigators (ZQL, MX and SLJ), based on previous research [12–15] and the guidelines published by the AAGBI and ASRA [8, 10]. The questionnaire consisted of 23 questions about the current RA practices and the availability of lipid emulsions in 2013. The survey responses were subsequently reviewed by four orthopedic anesthesiologists (XYG, XLW, LZ and ZGF) and an expert in LRT (XZX). The approved questionnaire is attached in Additional file 1.

The electronic questionaire was e-mailed to the 41 committee members of the orthopedic anesthesia group of the Chinese Society of Anesthesiology (CSA) on September 1st, 2013. Two follow-up e-mail reminders at 1-month intervals were sent to non-responders. December 1st, 2013 was the deadline to receive the feedback e-mails. Participation in this study was voluntary. Completion of the survey indicated a consent to study participation.

The respondents were grouped by the number of RA performed in patients undergoing orthopedic anesthesia per year, and we arbitrarily defined high-utilization institutions as those performing ≥ 50 % each year, and low-utilization institutions as < 50 % each year. Descriptive statistics were calculated. Fisher’s exact *t*-test was used for between-group comparisons. All statistical analysis was performed with SPSS for Windows (version 16.0; SPSS, Chicago, IL). A *p* < 0.05 was considered statistically significant.

## Results

### Survey response and RA utilization

Responses were received from 36 out of the 41 (88 %) anesthesiology departments; their characteristics are shown in Table 1. Out of the 36 institutions, 14 (39 %) used RA for orthopedic anesthesia in ≥ 50 % of patients per year and 22 (61 %) used RA for < 50 % of these patients. RA included PNBs, epidural blocks and spinal anesthesia.

**Table 1:** Characteristics of responding institutions

Hospital characteristics	Median (interquartile values)
Orthopaedics surgery (patient per year)	4000 (2500–7200)
Spine surgery	1000 (500–2000)
Upper and lower limb surgeries	2500 (1500–4800)
Percentage of orthopaedics surgeries performed under RA	30 % (16 %-70 %)

*RA* regional anesthesia, includes peripheral nerve blocks, epidural and spinal blocks

### RA practice

**Fig. 1:**
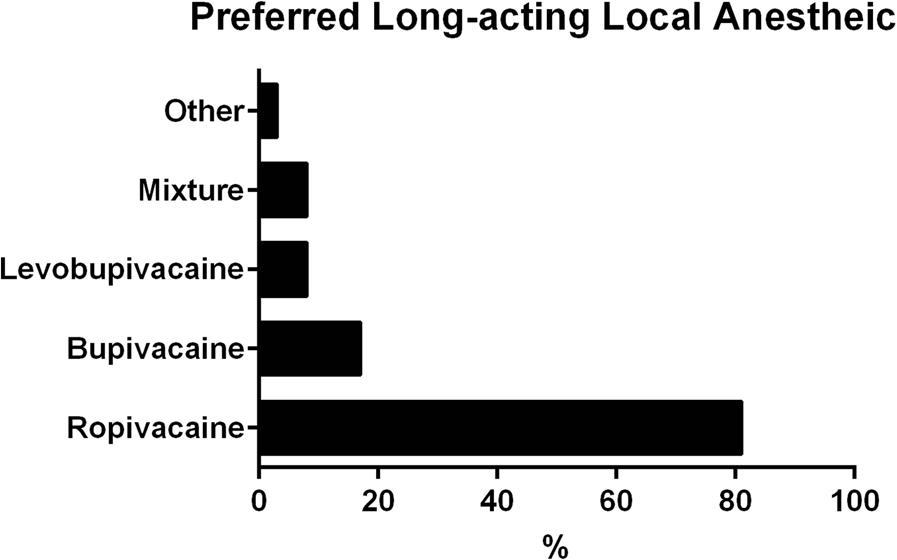
Long-acting local anesthetics preferred by responding institutions

RA was performed and monitored in the operating room of 29 (81 %) institutions, the RA induction area of 4 (11 %) institutions, and the postanesthesia care unit of 3 (8 %) institutions. With respect to the preferred local anesthetic when performing RA, 29 (81 %) respondents preferred ropivacaine and 6 (17 %) selected bupivacaine. There were 5 (14 %) responding departments selected multiple drugs as the preferred anesthetics. So the summation of all percentages exceeded 100 % (Fig. 1).

When performing RA, especially PNBs, almost all of the institutions stated that monitoring consisted of pulse oxygen saturation, noninvasive arterial blood pressure, and electrocardiogram. Only one institution reported that only pulse oxygen saturation was monitored during regional blocking. The distribution of the average number of PNBs performed monthly was shown in Table 2. In addtion, PNBs was performed by attending anesthesiologists at 28 (78 %) institutions and by senior anesthesiology residents at only 8 (22 %) institutions.

**Table 2:** Distribution of the average number of PNBs performed monthly in 2013

Number of PNBs	*n* (%)
0-10	4 (11 %)
11-30	6 (17 %)
31-60	14 (39 %)
>60	12 (33 %)

PNBs, peripheral nerve blocks

**Fig. 2:**
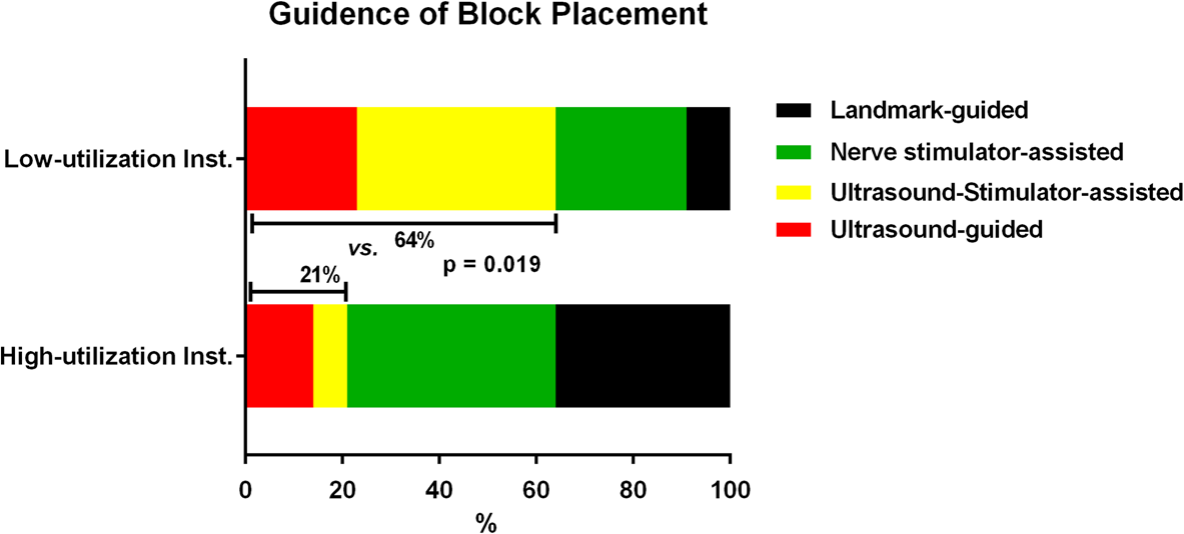
Techniques used by responding institutions for RA. The frequency of utilization of ultrasound differed significantly between high and low RA-utilization institutions (*P* = 0.019)

Regional blocking was performed under ultrasound guidance at 7 (19 %) institutions, under nerve stimulator guidance at 12 (33 %) institutions, under both techniques at 10 (28 %) institutions, and using landmark-based techniques at 7 (19 %) institutions. Interestingly, the use of ultrasound guidance by high- and low-utilization institutions differed significantly (21 % vs. 64 %, *P* = 0.019, Fig. 2).

### Incidence of LAST

Of the 36 responding institutions, 27 (75 %) reported an incident of LAST in 2013, among which, 14 respondents were high-utilization institutions while 13 respondents were low-utilization institutions. The incidence of LAST in the high-utilization institutions was much higher than that in the low-utilization institutions (100 % vs. 59 %, *P* = 0.006). The distribution of LAST among techniques used was: epidural anesthesia (31 %), brachial plexus block (50 %), cervical plexus block (23 %), lumbar plexus block (8 %), sciatic nerve block (4 %), combined lumbar and sciatic nerve block (31 %), and others (8 %).

Although 34 institutions (94 %) reported having an algorithm or guideline for the treatment of LAST, only the algorithms of 11 (31 %) of these institutions included lipid emulsions for treatment. All 36 responding institutions reported using lidocaine to detect intravascular epidural catheter placement, with five (14 %) adding epinephrine to the lidocaine.

### Adoption of lipid rescue

When respondents were asked about the adoption of LRT for management of LAST, 22 (61 %) knew that lipid emulsion infusion was a treatment option and 13 (36 %) had heard of LRT but did not know the specifics of the regimen. Eight institutions (22 %) were aware of the 2007 and 2010 AAGBI guidelines for LAST management [16, 17], 19 (53 %) were aware of the 2010 ARSA guidelines [8], and two (6 %) were aware of the 2011 ACMT guidelines [11].

### Lipid emulsion: availability and choice

**Fig. 3:**
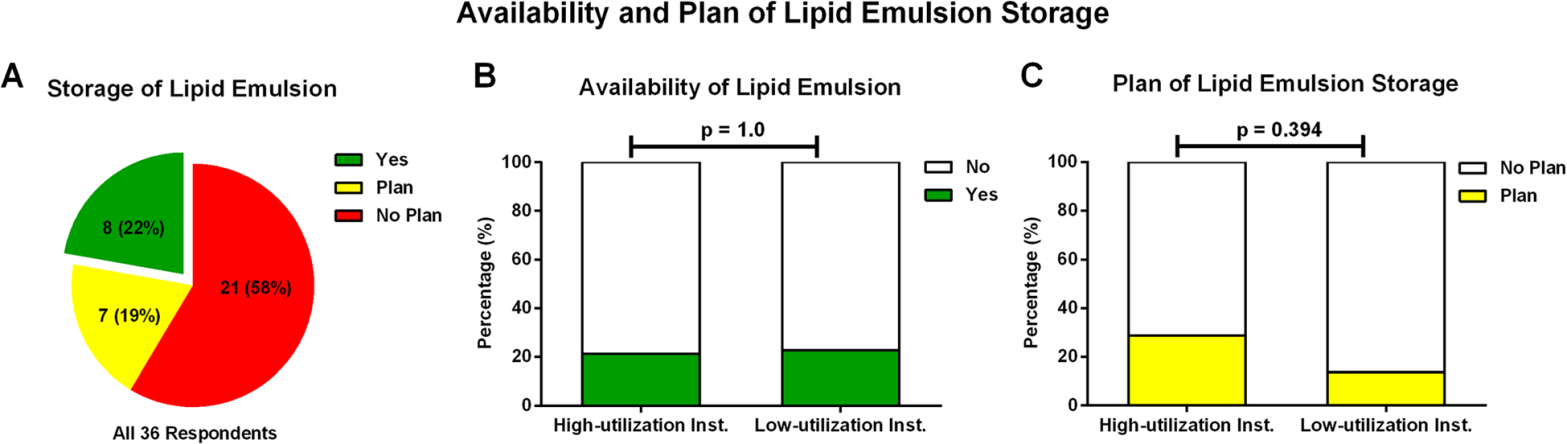
Adoption of lipid rescue therapy by responding institutions in China in 2013. **a** Current and future planned availability of lipid emulsion; **b** Current availability of lipid emulsion by high and low RA- utilization institutions (*P* = 1.0); **c** Future planned availability of lipid emulsion by high and low RA-utilization institutions (*P* = 0.394)

Lipid emulsion was readily available in 8 of the 36 (22 %) responding institutions (Fig. 3a). This availability was not associated with RA utilization (Fig. 3b). These eight institutions began to stock lipid emulsion between 2008 and 2012, with all eight institutions reporting storing lipid emulsion according to AAGBI [16, 17] and ASRA guidelines [8].

Of the 28 departments that did not currently stock lipid emulsion, seven (25 %) planned to stock it. The reasons for the remaining 21 departments that did not plan to stock lipid emulsion are shown in Table 3, with some departments citing more than one reason. In addition, high- and low-utilization institutions were similarly unlikely in planning to store lipid emulsion (Fig. 3c).

**Table 3:** Reasons reported for the unavailability of lipid emulsion in department of anesthesiology

Reasons	*n* (%)
Availability in center pharmacy	12 (50 %)
A low risk for LAST in the past years	7 (29 %)
Logistical reasons	3 (13 %)
View the LRT with suspicion	1 (4 %)
Unaware of the progress of LRT	1 (4 %)

*LAST* local anesthetic systemic toxicity, *LRT* lipid rescue therapy

The most common storage locations for lipid emulsion were anesthesia preparation rooms (4/8, 50 %) and a pharmacy within the department (3/8, 38 %). One respondent reported storing lipid emulsion in the postanesthesia care unit. All eight, however, reported that they could obtain the emulsion within 5 min for emergency treatment of LAST.

Of the eight units, five stocked lipid emulsions containing long-chain triglycerides (e.g. Intralipid; Huarui Pharmaceuticals Co., Ltd., Wuxi, China), two stocked lipid emulsions containing long- and medium-chain triglycerides (e.g. Lipovenoes; Huarui Pharmaceuticals Co., Ltd.); and one stocked both. Five of the eight units (63 %) reported using lipid emulsion to treat LAST within their departments. Of the 12 patients treated, 11 were successfully resuscitated; whereas one was not, making the overall failure rate of lipid resuscitation for LAST at 8.3 %.

### Resuscitation practice

When asked about treatment of ventricular tachycardia from presumed toxicity by local anesthetics, only five departments (14 %) reported they would use lipid emulsion. Of the remaining departments, 22 (61 %) would use amiodarone, five (14 %) would use lidocaine, and four (11 %) would use esmolol. Unfortunately, no data about the use of electrical cardioversion and its relation to the use of lipid emulsion in this questionnaire.

In response to convulsions secondary to LAST, only one respondent (3 %) reported that lipid emulsion would be the first-line treatment; whereas 25 (69 %) would choose a benzodiazepine, six (17 %) would use propofol, three (8 %) would use thiopental, and one (3 %) would use a muscle relaxant.

None of the 36 respondents reported using lipid emulsion for severe hypotension (MAP < 60 mmHg). In contrast, 20 (56 %) would consider epinephrine, 6 (17 %) would choose norepinephrine, five (14 %) would use dopamine and/or dobutamine, three (8 %) would use ephedrine, and two (6 %) would choose phenylephrine.

## Discussion

This survey showed that, from September to November 2013, lipid emulsion was available in only 22 % of academic anesthesiology departments listed by the orthopedic anesthesia group of the CSA. Of the remaining departments, only 25 % reported intention to stock lipid emulsion in the near future. The most common reason for not stocking lipid emulsion by the other 21 departments was “availability in the central pharmacy” within the hospital.

The long acting RA preferred by most departments was ropivacaine, followed by bupivacaine. However, bupivacaine is more cardiotoxic than similar agents. Thus, the greater safety of ropivacaine [18] may explain its widespread and, in some case, exclusive use by anesthesiologists in many institutions of China. Other factors, such as the cost of the agent and its duration of action, may also influence the choice by anesthesiologists. However, these factors were not evaluated in our study. Giving a test dose is a safety step to reduce LAST and was used by all institutions with 14 % of them using epinephrine-containing test solutions. Because other safety steps may not prevent intravascular injection, test dosing with an epinephrine-containing solution may have value. Any significant changes in heart rate and blood pressure may alert the anesthesiologist to intravascular injection of both epinephrine and anesthetic.

Over the past two decades, ultrasound-guided RA has been increasingly used by anesthesiologists worldwide. Compared with non-ultrasound techniques, ultrasound guided RA has been associated with reduced rates of inadvertent vascular puncture [19] and reduced LA requirements [20], resulting in reduction in the risk and severity of LAST [21]. Half of the respondents to our survey reported using ultrasound guidance and combined ultrasound and nerve stimulator guidance. Interestingly, ultrasound guided methods were more frequently used in low than in high RA-utilization institutions. The reasons for this difference remain unclear. Furthermore, the lower use of ultrasound guidance and higher incidence of LAST in high-utilization institutions may pointed to the clinical efficacy and safety of the performance of ultrasound-guided RA. Nevertheless, wether there is a negative correlation between use of ultrasound guidance and LAST or not is not presented in the current study and requires further research.

As LAST is a rare but devastating complication of RA, the availability of lipid emulsion is a patient-safety issue [14]. The AAGBI guidelines recommend that 20 % lipid emulsion should be “immediately available in all areas where potentially toxic doses of local anesthetics are administered.” All eight departments stocking lipid emulsion reported that they could obtain the drug within 5 min. However, many respondents that did not stock lipid emulsion reported that it was available at their central pharmacies. Thus, lipid emulsion was unlikely to be immediately available to those departments. Because AAGBI guidelines were not followed, increased physician awareness and education are warranted.

Our survey also assessed the effectiveness of lipid rescue in patients with LAST. Lipid resuscitation was successful in 11 of 12 events in the eight departments that store lipid emulsion. Although we did not assess the circumstances in which lipid emulsion was used, clinical and experimental reports have shown failure of lipid rescue in LAST management [22–24]. Failures may be due to the physicochemical properties of local anesthetics, an inadequate dose of lipid emulsion and/or interaction between the lipid emulsion and local anesthetics [25]. Optimal methods of administering lipid emulsion have not yet been determined.

The adoption of LRT in the United Kingdom was assessed in 2005–2008 by surveying 66 National Health Service hospitals within London and its surrounding areas [26]. Following the publication of the 2007 AAGBI guidelines [16], there was a sharp increase in the number of departments adopting these guidelines. By 2009, LRT was available in 95.1 % of institutions in England and Wales [15]. Moreover, two similar national surveys found that LRT was adopted in 2007 by 49 % of obstetric anesthesia units in the UK [13] and in 2011 by 88 % of obstetric anesthesia units in the United States [14]. Our survey found that the overall level of lipid emulsion availability was much lower, indicating the need to increase the awareness of LRT throughout China. Establishing Chinese national guidelines would likely contribute to this process.

This study had several limitations. As with any survey, it is subject to responder bias. However, we surveyed 41 academic hospitals that are distributed in all provinces and major municipalities in mainland China. In addition, our response rate was 88 %, suggesting that our results are as representative as possible. Also, we did not report incidents of local anesthetic toxicity with other fields as obstetric and peripheral vascular surgeries, in which lipid emulsion may be more available. However, orthopedic surgery in our study is the major indication for RA, with the utilization of PNBs in orthopedic surgery paralleling the increased number of ambulatory surgeries [27]. Thus, findings in hospitals performing orthopedic surgery likely reflect the adoption of LRT by academic anesthesiology departments. Finally, our results may not reflect the much broader practice of RA in non-academic departments throughout China.

## Conclusion

The survey found that LRT was available at few academic anesthesiology departments in China. The safety of perioperative RA can be optimized by increasing awareness of LAST and techniques for reducing and treating this condition. We encourage storage of lipid emulsion in specified locations, with current guidelines readily available. To date, no comparable survey has been reported in other developing countries. Our survey results may have been somewhat representative, thereby supporting efforts to globally promote LRT among practitioners.

## Additional file

Additional file 1:
**Survey questionnaire.** (DOC 53 kb)

## Abbreviations

RA: Regional anesthesia
LAST: Local anesthetic systemic toxicity
PNBs: Peripheral nerve blocks
LRT: Lipid rescue therapy
AAGBI: Association of anesthetists of Great Britain and Ireland
ASRA: American society of regional anesthesia
AHA: American heart association
ACMT: American college of medical toxicology
CSA: Chinese society of anesthesiology
