# Supplementary material for: Regional anesthesia and lipid resuscitation for local anesthetic systemic toxicity in China: results of a survey by the orthopedic anesthesia group of the chinese society of anesthesiology
Source: BMC Anesthesiol. 2016 Jan 4;16:1. doi: 10.1186/s12871-015-0163-0 (PMC4700758; doi:10.1186/s12871-015-0163-0)
Supplement: Additional file 1: — Survey questionnaire. (DOC 53 kb) [file 12871_2015_163_MOESM1_ESM.doc]

**Appendix 1. Survey questionnaire.**

| Please fill in the box of the appropriate answer:  Abbreviation: regional anesthesia, RA; peripheral nerve blocks, PNBs; local anesthetic systemic toxicity, LAST. | |
| --- | --- |
| 1. How many cases of orthopedic surgery you estimate are performed in your institution each year?   How many of those cases are upper limb surgeries? lower limb surgeries?  The percentage of regional anesthesia for those orthopedic surgery? % | |
| 1. Which one of the following long acting anethetics is used when performing regional anesthesia? | □ Ropivacaine □ Bupivacaine □ Levo-bupivacaine  □ Mixtures □ Other |
| 1. What about the average volume of PNBs performed monthly in your institution in 2013？ | □ 0-10 □ 11-30  □ 31-60 □ >60 |
| 1. Which one of the following techniques is most widely used when RA are performed in your unit？ | □ Ultrasound-guided block  □ Nerve stimulator-assisted block  □ Ultrasound-stimulator-assisted block  □ Landmark-guided block |
| 1. Who performs the majority of PNBs? | □ Postgraduate or fellow  □ Resident □Attending  □ Associate chief physician or senior |
| 1. Where are most PNBs performed in your unit? | □ Regional induction area □Postanesthesia care unit  □ Operating room  □ Somewhere else ( , please specify) |
| 1. What type of monitoring would the patients receive during the regional block procedure? | □ Pulse oximetry □ ECG  □ Pulse oximetry and noninvasive blood pressure  □ noninvasive blood pressure  □ Pulse oximetry and ECG  □ Pulse oximetry and noninvasive blood pressure and ECG |
| 1. Has there been any incident of LAST resulted from RA in 2013?   If “Yes”: what type of regional anesthesia did the patient receive at that time? | □Yes □ No  □ Epidural block □ Lumbar plexus block  □ Brachial plexus block □ Cervical plexus block  □ Sciatic nerve and lumbar plexus block  □ Sciatic nerve block  □ Other block ( , please specify) |
| 1. Does your unit have a test dose regimen designed to detect intravascular injection of local anesthetics?   If “Yes”, what local anesthetic is used in the test dose?  Does your unit use epinephrine as a part of test dose regimen? | □ Yes □ No  □ Lidocaine □ Lidocaine carbonate  □ Bupivacaine □ Ropivacaine  □ Yes □ No |
| 1. Does your unit have a guideline for the treatment of LAST?   If “Yes”, does this guideline include the use of intravenous lipid emulsion? | □ Yes □ No  □ Yes □ No |
| 1. Are you aware that lipid emulsion could be administered as a rescue medicine for LAST?   Do you know any following guidelines for the treatment of LAST with intravenous lipid emulsion? | □ Yes. I know the exact regimen of lipid emulsion for the treatment of LAST  □ Yes. But I don’t know how to use lipid for the treatment of LAST  □ No, I don’t know lipid could be used for the treatment of LAST  □ Guidelines for the Management of Local Anaesthetic Toxicity of AAGBI (2007/2010)  □ ASRA Practice Advisory on Local Anesthetic Systemic Toxicity (2010)  □ ACMT position statement: interim guidance for the use of lipid resuscitation therapy（2011） |
| 1. Are there lipid emulsion readily available for the treatment of LAST in your unit? | □ Yes (if “Yes”, please go to “13”)  □ No (if “No”, please go to “19”)  □ Planed (planed, please go to “20”) |
| 1. Where would you obtain the lipid emulsion? | □ Preparation unit □ Operating room  [□ Postanesthesia](../../../../E:%5C3%2520博士阶段开展的具体实验%5C实验5：Lipid%2520Rescue%2520AA%2520投稿%5CBMC%2520Anesthesiology%5CBMC%2520%2520一修%5C一修成品%5Cjavascript:void(0)%3B) care unit  □ Pharmacy in the department |
| 1. What type of lipid emulsions could you obtain? | □ Long-chain triglyceride emulsion  □ Long-and medium-chain triglyceride emulsion  □ Other type ( , please specify) |
| 1. How long would it take you to obtain the lipid emulsion according to the storage location in your unit? | □ ＜5 min □ 5-10 min  □ 10-30min □ ＞30min |
| 1. When did your unit begin to stock the lipid emulsion? | (Specific time, or indicate that closest to the time). |
| 1. What was the main reason for the decision to adopt lipid emulsion in your unit ? | □ Guidelines for the Management of Local Anaesthetic Toxicity of AAGBI (2007/2010)  □ ASRA Practice Advisory on Local Anesthetic Systemic Toxicity (2010)  □ Special Lecture from domestic and international academic conferences  □ Continued medical education |
| 1. Have you ever administered the lipid emulsion to treat LAST since the the availability of lipid emulsion in your unit? | □ Yes (If Yes, how many cases you succeed to treat? . Fail to rescue? )  □ No |
| 1. What is the exact reason for your unit not to stock the lipid emulsion? (not for the unit with storage of lipid emulsion) | □ Unaware of the progress of lipid rescue therapy  □ A low risk for LAST in the past years  □ View the LRT with suspicion  □ Availability in center pharmacy in hospital  □ Other reason ( , please specify) |
| 1. What type of lipid emulsion does your unit plan to stock? (not for the unit with storage of lipid emulsion) | □ Lipid emulsions containing long-chain triglycerides  □ Lipid emulsions containing long- and medium-chain triglycerides  □ Other type ( , please specify) |
| 1. What is your first choice in the treatment of seizure resulted from LAST? | □ Propofol □ Relaxants  □ Benzodiazepines □ Thiopental |
| 1. What is your first choice in the treatment of ventricular tachycardia resulted from LAST? | □ Amiodarone □ Verapamil  □ Lidocaine □ Esmolol |
| 1. What is your first choice in the treatment of severe hypotension (MAP<60mmHg) resulted from LAST? | □ Ephedrine □ Dopamine or Dobutamine □ Epinephrine □ Norepinephrine □ Milrinone or Amrinone  □ Other drugs ( , please specify) |
